# Impact of preoperative intraocular pressure on corneal endothelial cell loss after phacoemulsification in acute primary angle-closure glaucoma with cataracts

**DOI:** 10.3389/fmed.2025.1533950

**Published:** 2025-04-10

**Authors:** Jiaqi Shen, Xin Liu, Mingyue Lin, Yuting Shao, Guozhen Niu, Shen Qu, Yunli Niu, Qi Zhou, Li Zhang, Yanlong Bi

**Affiliations:** ^1^Department of Ophthalmology, Tongji Hospital, School of Medicine, Tongji University, Shanghai, China; ^2^Department of Ophthalmology, Guizhou Provincial People’s Hospital, Guiyang, China; ^3^Tongji Eye Institute, School of Medicine, Tongji University, Shanghai, China

**Keywords:** acute primary angle-closure glaucoma, phacoemulsification, ocular hypertension, corneal endothelial cell loss, endothelial cell density

## Abstract

**Purpose:**

This study aimed to assess corneal endothelial cell loss (ECL) following phacoemulsification and intraocular lens implantation (Phaco+IOL) in eyes with acute primary angle-closure glaucoma (APACG) and cataracts under different preoperative intraocular pressure (IOP) levels.

**Methods:**

This non-randomized controlled trial included 75 eyes from 75 patients with APACG and cataracts who underwent Phaco+IOL. All patients received pharmacotherapy and anterior chamber paracentesis before surgery and were grouped according to their preoperative IOP: the high-IOP group (IOP ≥ 25 mmHg) and the IOP-controlled group (IOP < 25 mmHg). IOP, visual outcome, endothelial cell density (ECD), hexagonality (HEX), coefficient of variation (CV), and central corneal thickness (CCT) were evaluated up to 3 months postoperatively. Baseline ECD, HEX, and CV parameters were measured in the contralateral eyes of all patients as a reference and compared with the postoperative results.

**Results:**

The average IOP decreased from 43.2 ± 4.8 mmHg to 16.5 ± 5.8 mmHg (*p* < 0.001) in the high-IOP group and from 18.3 ± 4.3 mmHg to 14.3 ± 3.2 mmHg (*p* < 0.001) in the IOP-controlled group on the first postoperative day. The changes in IOP were more significant in the high-IOP group (*p* < 0.001). The ECD at 3 months was 1705.2 ± 503.8 cells/mm^2^ in the high-IOP group and 2091.8 ± 330.1 cells/mm^2^ in the IOP-controlled group (*p* < 0.001). The ECL rates at 3 months were 35.0% (high-IOP group) and 17.4% (IOP-controlled group) (*p* < 0.001). The postoperative changes in HEX and CV at 3 months were more significant in the high-IOP group (*p* < 0.001; *p* = 0.003). Both groups produced comparable improvements in visual acuity and IOP.

**Conclusion:**

Uncontrolled high IOP (≥ 25 mmHg) before Phaco+IOL in patients with APACG and cataracts is associated with a higher rate of ECL. The rapid and substantial reduction of IOP during surgery may exacerbate corneal endothelial cell loss.

**Clinical trial registration:**

ClinicalTrails.gov, identifier ChiCTR2100052096.

## Highlights

- What is already known on this topic - Phacoemulsification has been established as an effective surgical intervention for IOP control in cases of acute angle-closure glaucoma secondary to anteriorly displaced and swollen lenses.
- What this study adds - Our study highlights that preoperative elevated IOP, combined with rapid and pronounced IOP reduction during phacoemulsification, may significantly exacerbate corneal endothelial cell injury. The potential mechanisms include mechanical trauma, alterations in the aqueous microenvironment, and inflammatory cascades precipitated by abrupt changes in IOP.
- How this study might affect research, practice, or policy - This study enhances the mechanistic understanding of factors underlying CEC loss during glaucoma management, emphasizing the need for adopting improved protective strategies during surgical interventions in patients with elevated IOP. The findings provide a foundation for future research and could inform clinical guidelines aimed at minimizing surgical risks and optimizing outcomes.

## 1 Background

Acute primary angle-closure glaucoma (APACG) is a sight-threatening ophthalmic emergency that is characterized by acute obstruction of the eye’s drainage angle and an increase in intraocular pressure (IOP) leading to optic nerve damage and visual field loss (1). Angle-closure glaucoma is estimated to affect 20 million people worldwide, with approximately three-quarters of cases concentrated in Asian populations (2, 3). In Asian populations, predisposing factors include shallow anterior chamber depth (ACD), short axial length, small corneal diameter, swollen lens, and weak zonules. Among these, a relatively swollen and more anteriorly positioned lens plays a crucial role in the mechanism of angle closure (4). With the refinement of surgical techniques and understanding of pathogenesis, phacoemulsification with intraocular lens implantation (Phaco+IOL) has been shown to be a safe and effective treatment for patients with APACG and cataracts, providing long-term IOP control (5, 6).

Corneal endothelial cells (CECs) are hexagonal, cuboidal cells located in the innermost layer of the cornea and play an important role in maintaining corneal transparency (7). The regenerative capacity of adult CECs is extremely limited. When CEC density decreases to critical levels, their ability to perform pump and barrier functions is compromised, followed by persistent edema and opacity of the cornea (7). Both glaucoma and phacoemulsification are established factors in corneal endothelial cell loss (ECL) (8, 9). When phacoemulsification is applied in glaucoma treatment, special attention should be given to the protection of the corneal endothelium (10–12). In particular, in cases where surgery is required under elevated IOP for patients with refractory APACG, a sudden intraoperative reduction in IOP may increase the risk of surgical complications. IOP fluctuations may also exert mechanical stress on the corneal endothelium, potentially contributing to its injury. This study aimed to observe the corneal endothelial cell status after Phaco+IOL surgery under varying IOP conditions, with a particular focus on elevated preoperative IOP, and to assess the possible causes that lead to endothelial cell loss.

## 2 Methods

### 2.1 Patients

This study is a monocentric, assessor-blinded, non-randomized controlled trial. We enrolled APACG patients with cataracts from August 2021 to June 2023 and conducted continuous follow-ups. The inclusion criteria are as follows: Patients were included if they presented with unilateral APACG coexisting with cataract, demonstrated an initial IOP ≥45 mmHg in the affected eye prior to treatment, and had an unaffected contralateral eye with clear corneal morphology and IOP <21 mmHg. The exclusion criteria are as follows: Patients were excluded if they had a history of glaucoma diagnoses (both angle-closure and other subtypes), corneal opacity or ocular fundus abnormalities, history of ocular trauma or intraocular surgical interventions, congenital ocular pathologies, a diagnosis of diabetes, or incomplete medical records. All eligible patients received 48 h of maximally tolerated medical therapy and anterior chamber paracentesis (fewer than 3 times) before surgery. After 48 h non-surgical treatments, 35 patients with persistent IOP above 25 mmHg were assigned to the high-IOP group, and 40 patients whose IOP was reduced to less than 25 mmHg were allocated to the IOP-controlled group. Institutional review board approval was obtained, and all patients provided informed consent. The study protocol conformed to the tenets of the Declaration of Helsinki.

### 2.2 Measurement

Demographic and baseline clinical characteristics were recorded. Anterior segment optical coherence tomography (AS-OCT; Carl Zeiss Meditec, Germany) and ultrasound biomicroscopy (UBM; MD-300 L, Tianjin Meda Co., Ltd.) were utilized to confirm the diagnosis and ensure that all included subjects were eligible for surgery (13). Routine examinations, including IOP, best corrected visual acuity (BCVA), and slit-lamp examination, were performed before and after surgery. The BCVA scores were converted to the logarithm of the minimum angle of resolution (logMAR). IOP was measured using a rebound tonometer (SW-500; Tianjin Soway). Central corneal thickness (CCT), endothelial cell density (ECD), hexagonality (HEX), and coefficient of variation (CV) were measured using a non-contact specular microscope (SP-1P; TOPCON, Japan). Since patients received corneal examination following the attack, severe corneal edema in the affected eye interfered with measurement. Therefore, ECD, HEX, and CV data from the contralateral eye were used as substitutes for the corneal endothelial status prior to the onset of APACG in all patients, serving as a baseline for comparison with the postoperative results in subsequent analyses. Measurements and analyses were performed separately by two technicians who were blinded to patient details.

### 2.3 Surgical technique

All surgeries were performed by the same experienced surgeon. The IOP was first rapidly reduced using a 25-gage needle to puncture the anterior chamber and drain the fluid. Subsequently, all patients underwent routine phacoemulsification with implantation of a posterior chamber foldable Aspira-aAY IOL (HumanOptics AG, Erlangen, Germany). Following this, 360°goniosynechialysis was performed under gonioscopic visualization. The viscoelastic used during the procedure was DisCoVisc® (Alcon, USA), which contains 1.65% sodium hyaluronate and 4% chondroitin sulfate. It exhibits both cohesive and dispersive properties, effectively maintaining the anterior chamber and providing protection to the corneal endothelium. Phacoemulsification was performed using a Centurion Vision System (Alcon Laboratories, Inc.) with an active-fluidic configuration. Nine eyes underwent additional anterior vitrectomy or irido-zonulo-hyaloid vitrectomy as indicated by intraoperative conditions. Specifically, six eyes in the high-IOP group and three eyes in the IOP control group required these interventions. In the high-IOP group, irido-zonulo-hyaloid-vitrectomy was performed in two cases to prevent the development of malignant glaucoma. All patients were followed up at 1 day, 1 week, 1 month, and 3 months after surgery.

### 2.4 Statistical analysis

Descriptive statistics are reported as the mean ± standard deviation (SD). For comparison of baseline characteristics, preoperative and postoperative parameters, and their differences across the two groups, continuous variables were analyzed using the independent Student’s *t*-test (with normal distribution) or Mann–Whitney *U*-test (without normal distribution), while the chi-square test was used for categorical variables. One-way ANOVA was used to assess temporal effects on IOP, BCVA, and ECD, followed by *post-hoc* multiple comparisons using the least significant difference (LSD) test. A *p*-value of <0.05 was considered statistically significant. The following standards were used: counting fingers = decimal acuity of 0.014 and detecting hand motion = decimal acuity of 0.005 (14).

## 3 Results

### 3.1 Baseline

A total of 75 affected eyes were enrolled in this study, including 35 eyes categorized in the high-IOP group and 40 eyes in the IOP-controlled group. All patients were of Han Chinese ethnicity. Demographic data and baseline clinical characteristics for both groups are summarized and compared in Table 1. Briefly, no statistically significant differences were observed between the two groups in terms of age, sex, lens thickness, axial length, anterior chamber depth, or the number of anti-glaucoma medications prescribed. Initial IOP and BCVA were assessed prior to the initiation of any treatment. The initial peak IOP was 68 mmHg in the high-IOP group and 65 mmHg in the IOP-controlled group.

**Table 1:** Demographic and baseline clinical characteristics of patients.

Variable	High-IOP group (*n* = 35)	IOP-controlled group (*n* = 40)	*p*-value
Age (y)	58.1 ± 12.0	60.3 ± 10.8	0.286^a^
Sex (male:female)	9:26	8:32	0.589^c^
lens thickness (mm)	4.29 ± 0.39	4.22 ± 0.41	0.165^a^
Axial length (mm)	21.7 ± 1.5	22.32 ± 1.2	0.364^a^
ACD (mm)	1.69 ± 0.36	1.82 ± 0.28	0.125^a^
Initial IOP (mmHg)	51.4 ± 4.9	50.6 ± 4.0	0.52^a^
Initial BCVA (logMAR)	1.52 ± 0.49	1.50 ± 0.31	0.451^b^
Number of anti-glaucoma drugs	3.1 ± 0.8	2.8 ± 0.7	0.27^b^
Phaco CDE	7.4 ± 1.1	7.6 ± 0.6	0.70^a^
Surgery time (min)	15.95 ± 4.95	14.28 ± 3.6	0.096^a^

LogMAR, logarithm of minimal angle of resolution.

a Independent *t*-test.

b Mann–Whitney *U* test.

c x^2^ test.

ACD, anterior chamber depth; IOP, intraocular pressure; BCVA, best corrected visual acuity; CDE, cumulative dissipated energy.

### 3.2 BCVA and IOP

Preoperative IOP and BCVA were measured 1 h prior to surgery, and the mean values before and after the procedure are summarized in Table 2. The IOP-controlled group demonstrated significantly better preoperative BCVA than the high-IOP group (*p* < 0.001). Postoperative BCVA at all time points showed significant improvement compared to preoperative values in both groups (*p* < 0.001). At the end of the follow-up period, there was no statistically significant difference in BCVA between the two groups.

**Table 2:** BCVA and IOP at various time points between the two groups.

		Preoperative	1 day	1 week	1 month	3 months
BCVA (logMar)	HIGH-IOP group	1.47 ± 0.32	0.89 ± 0.32^a^	0.65 ± 0.26^a^	0.54 ± 0.22^a^	0.41 ± 0.18^a^
	IOP-controlled group	0.98 ± 0.17^b^	0.72 ± 0.26^a^^b^	0.48 ± 0.20^a^^b^	0.41 ± 0.11^a^^b^	0.40 ± 0.16^a^
IOP (mmHg)	High-IOP group	43.2 ± 4.8	16.5 ± 5.8^a^	16.7 ± 4.5^a^	15.1 ± 3.6^a^	14.7 ± 4.3^a^
	IOP-controlled group	18.3 ± 4.3^b^	14.3 ± 3.2^a^^b^	12.6 ± 3.9^a^^b^	13.9 ± 3.8^a^^b^	15.2 ± 3.2^a^

a Compared to preoperative, the difference was statistically significant (*p* < 0.05).

b At the same time point, there were significant differences between the two groups (*p* < 0.05).

**Figure 1:**
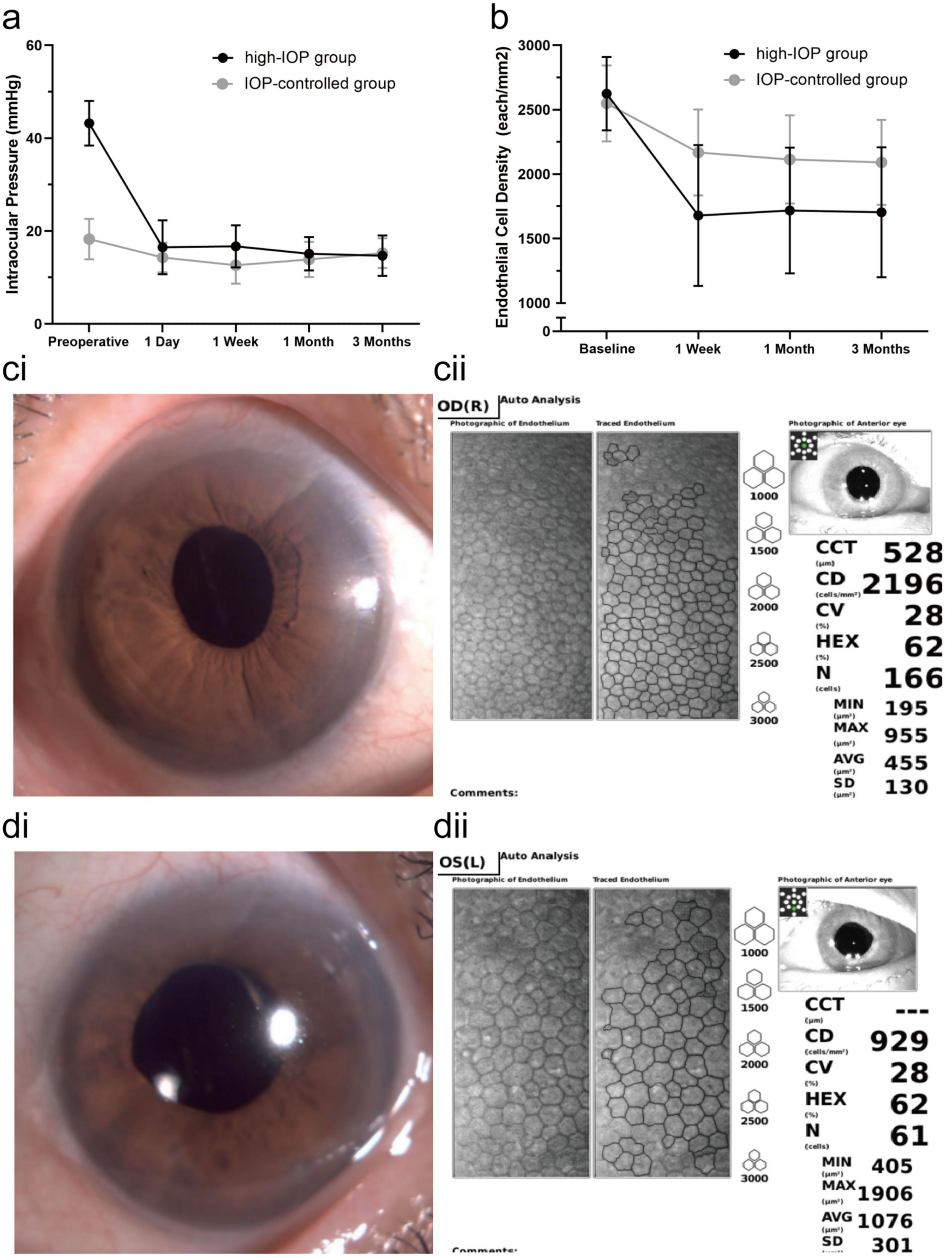
**(a)** Change trend of IOP in the high-IOP group and the IOP-controlled group before and after surgery. **(b)** Change trend of ECD in the high-IOP group and IOP-controlled group before and after surgery. Error bars represent standard deviation. **(c)** The cornea of the patient in the IOP-controlled group after surgery: **(ci**,**di)** Corneal state under slit-lamporneal endothelium. **(cii**,**dii)** CECs were determined by a non-contact specular microscope and a computer-aid image analysis system.

The mean preoperative IOP in the high-IOP group was elevated due to insufficient response to non-surgical treatments. Of these, 14 patients had an IOP below 40 mmHg, 18 patients had an IOP between 40 and 60 mmHg, and 3 patients had an IOP above 60 mmHg. In contrast, preoperative IOP in the IOP-controlled group was reduced to 18.3 ± 4.3 mmHg (12 ~ 25 mmHg) (*p* < 0.001) by non-surgical treatment. Of these, 28 patients had an IOP below 21 mmHg, and 12 patients had an IOP between 21 and 25 mmHg. On the first postoperative day, both groups experienced significant reductions in IOP (*p* < 0.05). However, the intraoperative reduction in IOP was more pronounced in the high-IOP group compared to the IOP-controlled group (26.7 ± 5.3 mmHg vs. 3.9 ± 4.1 mmHg; *p* < 0.001). At the 3-month follow-up, no significant difference in mean IOP was observed between the two groups (*p* = 0.545). The trends in BCVA and IOP changes over time for both groups are illustrated in Figure 1a.

### 3.3 Characteristics of corneal endothelial cells

Baseline corneal endothelial parameters and CCT showed no statistically significant differences between the two groups. The mean ECD and ECL rates at various time points for both groups are summarized in Table 3. One week after surgery, ECD decreased significantly in both groups (*p* < 0.001), with a more pronounced decrease in the high-IOP group (*p* < 0.001). At the end of follow-up, the ECL rate was 35.0% in the high-IOP group compared to 17.4% in the IOP-controlled group (*p* < 0.001). The decreasing trends in ECD in the two groups are shown in Figure 1b.

**Table 3:** Comparison of ECD at various time points between the two groups.

	Baseline	1 week	1 month	3 months
high-IOP group				
ECD (cells/mm^2^)				
Mean ± SD	2625.2 ± 284.5	1680.4 ± 546.6^a^	1719.0 ± 488.2^a^	1705.2 ± 503.8^a^
Min-Max	2016–2930	632–2539	618–2535	654–2473
Change from baseline	–	945.1 ± 585.4	909.2 ± 540.5	919.9 ± 573.3
ECL rate (%)	–	36.0%	34.6%	35.0%
IOP-controlled group				
ECD (cells/mm^2^)				
Mean ± SD	2550.1 ± 295.2	2168.8 ± 333.3^a^^b^	2115.1 ± 342.6^a^^b^	2091.8 ± 330.1^a^^b^
Min-Max	1982–3025	1328–2845	1069–2605	1135–2686
Change from baseline	–	367.7 ± 274.3^c^	421.4 ± 311.1^c^	444.7 ± 324.2^c^
ECL rate (%)	–	14.4%	16.5%	17.4%

a Compared to baseline parameters within the group *p* < 0.05.

b Comparison between the two groups at the same time point, *p* < 0.05.

c The changes in ECD from baseline between the two groups were compared, *p* < 0.05.

ECD, endothelial cell density; ECL, endothelial cell loss.

The changes in CCT, HEX, and CV at baseline and post-surgery are shown in Table 4. Prior to treatment, corneal edema was evident in both groups. Postoperatively, the corneal edema subsided within 1 week. CCT was similar in both groups at 3 months (*p* = 0.129). There were no significant differences in the HEX and CV of the contralateral eyes between the two groups at baseline (*p* = 0.174; *p* = 0.615). Three months postoperatively, significantly greater changes were observed in the HEX and CV of the affected eyes in the high-IOP group compared to the IOP-controlled group (*p* = 0.003; *p* = 0.002). Relative to the baseline, the average HEX and CV changed more significantly in the high-IOP group (*p* < 0.001; *p* = 0.003). Representative images of corneal endothelial characteristics from the two groups are illustrated in Figure 1. In the IOP-controlled group (Figure 1c), the cornea remained transparent under slit-lamp examination (Figure 1ci), with only mild endothelial damage observed (Figure 1cii). In contrast, in the high-IOP group (Figures 1di,dii), marked corneal edema and significant ECD reduction were evident.

**Table 4:** Comparison of corneal endothelial morphology and CCT data between two groups.

		Baseline*	3 months	Change
HEX (%)	High-IOP group	55.5 ± 4.3	49.3 ± 6.4^a^	6.1 ± 3.5
	IOP-controlled group	56.7 ± 3.6	53.2 ± 5.8^a^^b^	3.5 ± 2.9
				*t* = 3.57, *p* < 0.001
CV (%)	High-IOP group	32.1 ± 3.8	37.3 ± 4.2^a^	5.2 ± 3.7
	IOP-controlled group	31.7 ± 4.4	34.2 ± 3.9^a^^b^	2.5 ± 3.1
				*t* = 3.08, *p* = 0.003
CCT (μm)	High-IOP group	628.9 ± 33.6	582.3 ± 25.9^a^	46.6 ± 9.4
	IOP-controlled group	619.4 ± 23.8	577.4 ± 26.2^a^	43.0 ± 8.1
				*t* = 12.42, *p* = 0.129

a Compared to baseline parameters within the group, *p* < 0.05.

b Comparison between the two groups at the same time point, *p* < 0.05.

*Baseline HEX and CV are values of the fellow eyes. Baseline CCT was obtained from attacked eyes before the patients received any treatment.

### 3.4 Postoperative pharmacotherapy and complications

Postoperatively, only four eyes (11.4%) in the high-IOP group required 1–2 types of anti-glaucoma medications to assist with IOP control. Among them, three or four clocks of peripheral anterior synechia were observed in two eyes with IOP elevated to 25 mmHg, which was managed with medication. Tobramycin-dexamethasone eye drops (TobraDex, S.A., Alcon Couvreur N.V., Belgium) were administered four times daily to all patients to reduce inflammation. Eleven eyes (31.4%) in the high-IOP group and two eyes (5%) in the IOP-controlled group exhibited significant anterior chamber inflammation (*p* < 0.001). For these patients, steroid frequency was increased, or periocular steroid injections were administered to resolve inflammation within 1 week. A total of 31 eyes (88.6%) in the high-IOP group and 3 eyes (7.5%) in the IOP-controlled group developed dilated and fixed pupils, which likely contributed to suboptimal postoperative visual acuity. No other complications occurred, including persistent corneal edema, posterior capsule rupture, intraocular lens dislocation, or suprachoroidal hemorrhage.

## 4 Discussion

Corneal endothelium damage in glaucoma is influenced by various factors, including elevated IOP, direct iris-cornea contact, alterations in the aqueous microenvironment, surgical interventions, and associated inflammatory responses (9, 15). High IOP is considered the primary factor affecting CECs through three mechanisms: direct mechanical damage, impaired pump function, and ischemic oxidative stress (15, 16). Notably, both the severity and duration of IOP elevation are positively correlated with ECL (15, 17). For patients with high IOP refractory to conservative therapy, surgical intervention is recommended to prevent irreversible optic nerve damage. Although phacoemulsification is an effective method for experienced surgeons to reduce IOP in patients with APACG, surgery under high IOP has been widely recognized as high risk and prone to complications (11, 18, 19). Corneal endothelial cells are particularly vulnerable in this context, facing considerable challenges during surgery (20). Our study compared two cohorts of APACG patients with varying preoperative IOP control to assess phacoemulsification efficacy and postoperative corneal endothelial cell loss.

In this study, the IOP in the high-IOP group decreased from 43.2 ± 4.8 mmHg preoperatively to 16.5 ± 5.8 mmHg on the first postoperative day. By contrast, in the IOP-controlled group, the IOP first decreased to 18.3 ± 4.3 mmHg by pharmacological intervention. This reduction process was gradual in nature, as the decrease in IOP through pharmacotherapy is generally slow. Subsequent surgical intervention further decreased IOP to 14.3 ± 3.2 mmHg. Notably, intraoperative changes in IOP were more pronounced and abrupt in the high-IOP group compared to the IOP-controlled group. ECL was significantly higher in the high-IOP group (35.0% vs. 17.4%, *p* < 0.001), alongside marked endothelial cell morphological changes.

ECL in the IOP-controlled group was consistent with previous studies. Kubota et al. found that ECL was approximately 18.3% after Phaco+IOL in angle-closure glaucoma patients whose IOP had been controlled by iridotomy beforehand (12). Phacoemulsification has been associated with ECL, with contributing factors including nuclear density, phacoemulsification time/energy, lens fragment contact, and fluid turbulence. The Centurion Vision System was utilized, which reduces cumulative dissipated energy (CDE), aspiration volume, and surgical time while enhancing efficiency (21). Moreover, the Centurion equipped with a control system can achieve continuous and stable anterior chamber pressure to relieve intraocular trauma and protect the CECs (22). Notably, in our study, intraoperative CDE demonstrated relatively low values in both groups (7.4 ± 1.1 and 7.6 ± 0.6, respectively), with no significant difference between the two groups. Additionally, the total surgical duration—from initial incision to wound closure—was analyzed. The operative times (15.95 ± 4.95 and 14.28 ± 3.6 min, respectively) also revealed no significant intergroup differences. Therefore, phacoemulsification was insufficient to explain the difference in ECL between the two groups.

We hypothesized that the higher rate of ECL in the high-IOP group could be attributed to mechanical damage and aqueous environment alterations induced by a sudden drop in intraoperative IOP (10). Sudden and drastic changes in IOP may destabilize the external physical environment of CECs, thereby compromising their structural integrity (23). Rapid aqueous humor release through the corneal incision generated shear forces, potentially causing mechanical detachment of CECs and inducing apoptosis (19, 23). Rapid IOP reduction could further compromise the permeability of vascular tissues in the anterior chamber vasculature (24). Furthermore, the postoperative inflammatory response was more pronounced in the high-IOP group, which altered aqueous humor composition, contributing to CEC damage (10, 11).

To protect CECs, we recommend a stepwise approach to IOP reduction, particularly for patients with uncontrolled APACG. Gradual intraoperative drainage of aqueous humor or anterior vitrectomy may allow CECs to better adapt to changes in pressure (25). Noh et al. demonstrated that performing a limited pars plana vitrectomy as an initial step to reduce IOP is more effective in preserving CECs compared to direct phacoemulsification in patients with uncontrolled high IOP (25).

In this study, the initial corneal examination was affected by corneal edema in the affected eye. Therefore, baseline ECD, HEX, and CV were obtained from the unaffected contralateral eye in all patients (26). We excluded patients with a history or suspected history of glaucoma, intraocular surgery, intraocular inflammation, infection, ocular trauma, hereditary eye diseases, or diabetes prior to this episode. Since the CECs of individuals without these factors are recorded as symmetric (27–29), it is acceptable to use the parameters from the contralateral eye as a substitute for the corneal endothelial cell state before the onset of APACG in this study. Moreover, while APACG typically presents in one eye, the anatomical similarities between the two eyes (1, 30) make the contralateral eye a more reliable reference than using parameters from normal eyes of the same age group.

A limitation of our study is the relatively small sample size (*n* = 75). Additionally, anterior chamber paracentesis was conducted in some patients. Although this procedure was performed only when strictly necessary, with the number of punctures restricted to fewer than three, it may still have influenced the corneal endothelium. Currently, robust supporting evidence in the literature is lacking, underscoring the need for further investigation.

In conclusion, our study suggests that Phaco+IOL is effective for treating APACG with poorly controlled IOP. However, rapid and pronounced reductions in IOP during surgery appear to significantly contribute to ECL *in vivo*. These findings underscore the importance of carefully managing IOP fluctuations during surgery to minimize corneal endothelial damage and optimize patient outcomes.

## Data Availability

The raw data supporting the conclusions of this article will be made available by the authors, without undue reservation.
